# Ethylene Glycol Ethers Induce Oxidative Stress in the Rat Brain

**DOI:** 10.1007/s12640-014-9486-8

**Published:** 2014-08-02

**Authors:** Bartosz Pomierny, Weronika Krzyżanowska, Irena Smaga, Lucyna Pomierny-Chamioło, Piotr Stankowicz, Bogusława Budziszewska

**Affiliations:** 1 Department of Biochemical Toxicology, Medical College, Jagiellonian University, Medyczna 9, 30-688 Kraków, Poland; 2 Department of Toxicology, Medical College, Jagiellonian University, Medyczna 9, 30-688 Kraków, Poland; 3 Department of Experimental Neuroendocrinology, Institute of Pharmacology, Polish Academy of Sciences, Smętna 12, 31-343 Kraków, Poland

**Keywords:** Ethylene glycol ethers, Neurotoxicity, Antioxidant enzymes, Lipid peroxidation

## Abstract

Ethylene glycol ethers (EGEs) are components of many industrial and household products. Their hemolytic and gonadotoxic effects are relatively well known while their potential adverse effects on the central nervous system have not yet been clearly demonstrated. The aim of the present study was to examine the effects of 4-week administration of 2-buthoxyethanol (BE), 2-phenoxyethanol (PHE) and 2-ethoxyethanol (EE) on the total antioxidant capacity, activity of some antioxidant enzymes, such as the superoxide dismutase (SOD), catalase, glutathione peroxidase (GPX) and glutathione reductase and lipid peroxidation in the frontal cortex and hippocampus in the rat. These studies showed that BE and PHE decreased the total antioxidant activity, SOD and GPX activity, while increased lipid peroxidation in the frontal cortex. Like in the frontal cortex, also in the hippocampus BE and PHE attenuated the total antioxidant activity, however, lipid peroxidation was increased only in animals which received BE while reduction in GPX activity was present in rats administered PHE. The obtained data indicated that 4-week administration of BE and PHE, but not EE, reduced the total antioxidant activity and enhanced lipid peroxidation in the brain. In the frontal cortex, adverse effects of PHE and BE on lipid peroxidation probably depended on reduction in SOD and GPX activity, however, in the hippocampus the changes in the total antioxidant activity and lipid peroxidation were not connected with reduction of the investigated antioxidant enzyme activity.

## Introduction

Ethylene glycol ethers (EGEs) are ingredients of many industrial, household and pharmaceutical products. EGEs possess advantageous physicochemical properties, like low vapor pressure, solubility in ethanol and water mixtures which makes them a superb solvent with multiple applications (Boatman and Knaak 2001). Hence, they are used in cooling liquids, varnishes, pesticides, herbicides, household liquids, vaccines or antiseptic specifics and many others. The most broadly known EGEs include: 2-methoxyethanol (ME), 2-ethoxyethanol (EE), 2-buthoxyethanol (BE), 2-propoxyethanol (PE), 2-isopropoxyethanol (IPE) and 2-phenoxyethanol (PHE). EGEs are well absorbed following dermal, inhalation or oral exposures and are quickly distributed throughout the body (Hardin et al. 1984; Ku et al. 1995; Lockley et al. 2002; Udden 2000). Many studies, both, in vitro and in vivo have shown that these compounds exert toxic influence on reproductive, developmental, immunological and hematological systems (Barbee et al. 1984; Johanson 2000; Lamb et al. 1984; Starek et al. 2008a; Williams et al. 1995). It has been proven that in vivo EGEs are metabolized mainly via oxidation by alcohol dehydrogenase to alkoxyacetaldehyde and next are converted by aldehyde dehydrogenase to alkoxyacetic acid (Ghanayem et al. 1987; Ma et al. 1993). Toxic effect of EGEs in the periphery is exerted predominantly by their metabolites, mainly alkoxyacetic acids (Ghanayem and Sullivan 1993). Short-chain EGEs, e.g. ME and EE have predominantly gonadotoxic effect. In male rats, ME and EE disrupted testicular function by causing degeneration of spermatocytes and reduction of the number of spermatocytes and spermatids in the experimental model of intoxication (Adedara and Farombi 2010; Lamb et al. 1984). In female rats, these compounds caused toxic effect mainly on ovarian luteal cells (Davis et al. 1997). People with occupational exposure to EE and ME suffered from disturbance in menstrual cycle in women and reduction in sperm count in men (Gold 1995; Welch 1988). Contrary to ME and EE, BE and IPE exert a potent hemolytic effect. Such effect has been reported not only in experimental animals, but also in clinical observations of patients intoxicated with BE or IPE (Ghanayem and Sullivan 1993; Starek et al. 2008a, b). These EGEs significantly reduced the red blood cell, thrombocyte and leukocyte counts and hemoglobin level. It has been also reported that these compounds caused bone marrow suppression (Haufroid et al. 1997; Kim et al. 1999) and exerted toxic effects on the immune system.

The toxic effects of EGEs on the hematopoietic system, reproductive system, immune system are relatively well-described, whereas the studies of a potential neurotoxicity of these compounds are very scanty, yet. Clinical observations of intoxicated patients showed that EGEs affected brain function and depending on the dose caused headache, impairment of cognitive function, disturbed motor coordination, convulsions or central nervous system depression (Ahmed et al. 1983; Morton 1990; Ohi and Wegman 1978). It is known that EGEs cross the blood–brain barrier, so their adverse effect on the CNS and involvement in triggering and progress of neurodegenerative changes is highly probable. Previously in in vitro experiments we found that BE, PHE and IPE more severely than ME or EE damaged human neuroblastoma cells (SH-SY5Y). Those experiments indicated that more lipophilic EGEs like BE or PHE are more harmful to neurons than those EGEs with higher hydrophilic properties (Regulska et al. 2010). In the next experiment, we showed that also in vivo a mixture of two EGEs induced adverse reactions in the brain by decreasing the total antioxidant capacity, enhancing lipid peroxidation and increasing caspase-3 activity in the rat hippocampus and frontal cortex (Pomierny et al. 2013). However, in that study we examined the action of combined administration of two EGEs, with different chain lengths, since industrial and household products most often contain EGE mixture.

The aim of the present study was to examine a potential toxic effect of BE, PHE and EE on the rat hippocampus and frontal cortex. Since oxidative stress is considered to be a major factor involved in induction of nerve cell damage, the effect of EGEs on the total antioxidant capacity, activity of antioxidant enzymes (superoxide dismutase, catalase, glutathione peroxidase and glutathione reductase) and lipid peroxidation was determined. PHE was chosen for this study, because this compound exerted the most potent cytotoxic effect on SH-SY5Y cells and its potential neurotoxic activity has not been investigated so far, either alone or in a mixture, in in vivo models. Moreover, phenoxyacetic acid, a PHE metabolite, occurs in the urine of general population and after exposure to preparations containing PHE during cleaning procedures its concentration is the highest, and, therefore, it creates the greatest risk (Fromme et al. 2013). BE was selected because it strongly enhanced oxidative stress-induced damage of SH-SY5Y cells and administered to rats in a mixture with ME or EE evoked oxidative stress in the brain. Moreover, production of BE has increased in recent years to replace gonadotoxic ME and EE and now more than a half of household preparations contains BE. Contrary to PHE and BE, which have a clearly lipophilic character and, therefore, are more likely to pass into the brain, EE is more hydrophilic and should exert a weaker effect in the brain. However, we included it in this study, because there are no experimental data concerning its level and action in the brain, so its potential adverse effects cannot be excluded and additionally the mechanism of action of this compound in peripheral tissues is the best known, so far. For example, it has been found that EE decreased glutathione level, superoxide dismutase and catalase activity and enhanced lipid peroxidation in the rat testes (Adedara and Farombi 2010), so it potentiated oxidative stress, crucially involved also in brain cell damage. The potential adverse effect of 4-week administration of the selected EGEs on oxidative stress markers and lipid peroxidation was evaluated in the hippocampus and frontal cortex since these brain region are more susceptible to damage.

## Materials and Methods

### Animals

The experiments were performed on male Wistar rats (250–330 g). The animals were kept under natural day-night cycle, at 22 ± 2 °C with food and water available at libitum. All procedures were conducted according to the NIH Guide for the Care and Use of Laboratory Animals and were approved by the Local Ethics Committee. The rats were randomly divided into five groups of eight animals each (*n* = 8). Animals were treated subcutaneously with saline, EE (2.5 mM/kg), BE (2.5 mM/kg), sunflower oil and PHE (2.5 mM/kg) once a day, 5 days per week, for 4 weeks. Due to the solubility of PHE in the sunflower oil, there were two control groups—receiving saline or sunflower oil. All the solutions were sterile and neutralized to pH of 7.4. The volume of solution administered to each animal was in the range of 250–330 µl as the solution was prepared at the concentration of 2.5 mM/ml. The rats were treated with the examined compounds 5 days per week in order to model workers’ exposure. We did not notice any escalated behavioral pain response after first as well as subsequent injections. After animals were sacrificed we examined subdermal tissue in the place of injections (lower middle part of the groin) to any signs of inflammation, with negative results.

It should be noted that stress connected with repeated, subcutaneous administration of EGEs can modulate the activity of investigated antioxidant enzymes and lipid peroxidation, so the effect of test compounds was compared to appropriate parameters obtained in animals receiving the solvent alone, respectively saline or oil, and subjected to the same procedures as animals injected with compounds tested.

The animals were decapitated 24 h after the last injection and their brains were quickly removed from the skull. The frontal cortex and hippocampus were isolated at low temperature (on ice) and immediately frozen on dry ice and stored at −80 °C until they were used for biochemical assays. Tissues were dissected out according to The Rat Brain Atlas (G. Paxinos, C. Watson, The Rat Brain in Stereotaxic Coordinates, fourth ed., Academic Press, San Diego, 1998). Frozen brain tissues were homogenized in 50 mM phosphate buffer solution (pH = 7.4).

Each control and experimental groups contained eight samples and each sample was determined in triplicates.

### Total Antioxidant Capacity (TAC)

Analysis of TAC was carried out on the brain structure homogenates according to the modified method of Benzie and Strain (1996) adapted to specific tissue and microplate assay. The tissue antioxidant ability was evaluated by reduction of ferric ions. Fe(III)–tripyridyltriazine (Fe(III)–TPTZ) complex is reduced to blue Fe(II)–tripyridyltriazine (Fe(II)–TPTZ), the level of which was determined spectrophotometrically at 573 nm. The absorbance was measured by a multiwell plate reader (TECAN Infinite M200). The total antioxidant capacity of samples was calculated from the standard curve and was expressed as mM Fe(II) per mg of protein.

### Superoxide Dismutase (SOD) Activity

The assay uses the xanthine/xanthine oxidase (XOD) system as a source of superoxide anions which reduce the nitroblue tetrazolium salt to a water-soluble formazan. The method is based on the principle, that SOD decomposes superoxide anions and thus inhibits formazan formation. The chromogen level in the sample was determined spectrophotometrically with absorption maximum at 560 nm. Since the level of SOD in the sample is proportional to the assay reaction inhibition, the results are expressed as the inhibition rate in %.

### Catalase (CAT) Activity

Catalase activity was determined by Catalase Assay Kit (Cayman, US). The method is based on the reaction of tissue homogenate catalase in the presence of methanol and optimal concentration of H_2_O_2_. The product of this reaction, which is formaldehyde, reacts with a specific chromogen to form purple heterocyclic compound with maximal absorbance at 540 nm. Catalase activity in samples was calculated from the standard curve and was expressed as U/mg of protein.

### Glutathione Peroxidase (GPx) Activity

The activity of GPx was measured indirectly by a coupled reaction with glutathione reductase by GPx Assay Kit (Cayman, US). In this method, GPx reduces cumene hydroperoxide during oxidation of GSH to GSSG. At this moment, the total sample glutathione level is equal to GSSG generated by addition of cumene hydroperoxide. Next, the generated GSSG is reduced again to GSH by glutathione reductase with consumption of NADPH. The decrease in NADPH is proportional to the activity of GPx. NADPH is quantified at the maximal absorbance at 340 nm. The assay was conducted on the fresh tissue homogenate at 37 °C, and the decrease in absorbance was monitored for 7 min. The absorbance was measured by a multiwell plate reader TECAN Infinite M200. The results of this assay are presented as nmol NADPH/min/mg protein.

### Glutathione Reductase (GR) Activity

The activity of GR was determined by GR Assay Kit (Cayman, US). The assay is based on the reaction catalyzed by GR, in which GSSG is reduced to GSH, in turn NADPH is oxidized to NADP+. The oxidation of NADPH is accompanied by a decrease in absorbance at 340 nm and is directly proportional to the activity of GR in the sample. The assay was conducted on the fresh tissue homogenate The reaction was initiated by addition of NADPH into the mixture of sample and GSSG and then the measurement of absorbance at 340 nm was conducted for 5 min. The results are presented as nmol NADPH/min/mg protein.

### Lipid Peroxidation (MDA) Level

Colorimetric assay for lipid peroxidation was performed using LPO-586 test (Bioxytech S.A.). The assay is based on the reaction of the chromogenic reagent, *N*-methyl-2-phenylindole, with one of the main products of the lipid peroxidation malondialdehyde (MDA) at 45 °C. The reagent produces a violet 586 nm absorbing pigment with a trimethine structure, a stable chromophore with maximal absorbance at 586 nm, in hydrochloric acid. This type of assay is more reliable than the reaction with tiobarbituric acid in which heating up to 100 °C is performed what can induce de novo production of MDA and other products of lipid peroxidation. The absorbance was measured by a multiwell plate reader TECAN Infinite M200. Lipid peroxidation in samples was calculated from the standard curve and is expressed as nmol of MDA/mg of protein.

### Protein Concentration

Protein concentration was determined in the brain structure homogenates using bicinchoninic acid (Smith et al. 1985). This method combines reduction of Cu^+2^ to Cu^+1^ by protein in an alkaline medium (the biuret reaction) with the colorimetric detection of the cuprous cation (Cu^+1^) using the bicinchoninic acid. The purple-colored reaction product of this assay is formed by the chelation of two molecules of BCA with one cuprous ion. This water-soluble complex exhibits a strong absorbance at 562 nm, which is linear with increasing protein concentrations. The absorbance was measured by a multiwell plate reader TECAN Infinite M200.

### Statistical Analysis

All data were expressed as the means (±SEM) from eight samples assayed in triplicates. Statistical analyses were performed with a one-way analysis of variance (ANOVA) and next differences between groups were evaluated by the Dunnett’s post-hock test. *p* < 0.05 was considered as statistically significant.

## Results

### Total Antioxidant Capacity

**Fig. 1:**
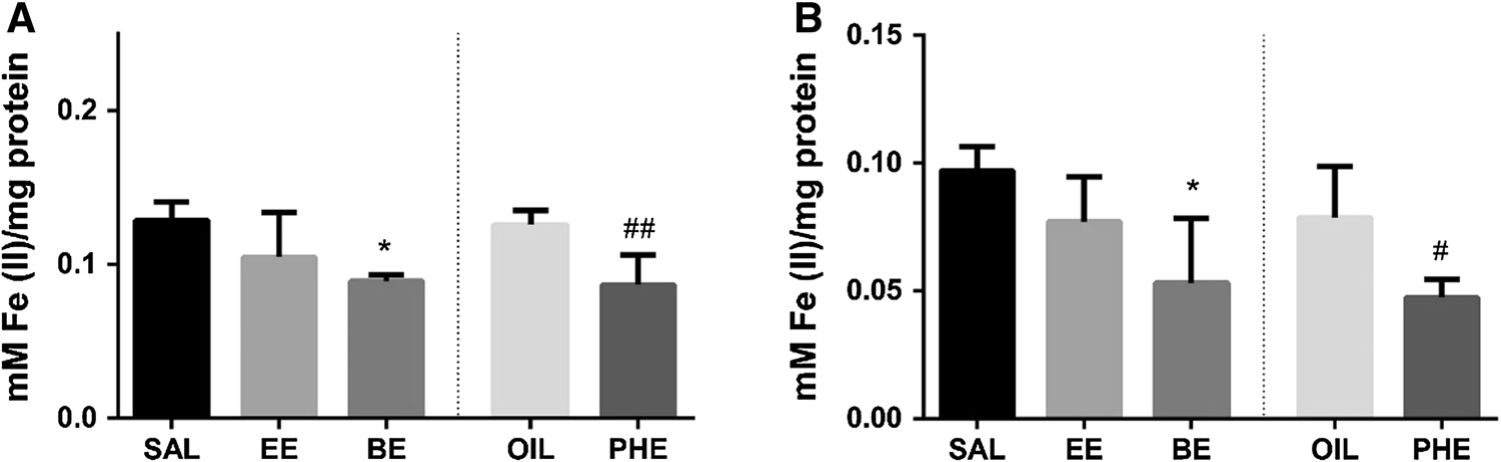
The effect of EGE on the total antioxidant capacity in the frontal cortex (**a**) and hippocampus (**b**). The results are shown as the mean ± SEM from 8 tissue samples run in three repetitions. *SAL* saline; *EE* 2-ethoxyethanol; *BE* 2-butoxyethanol; *OIL* sunflower oil; *PHE* 2-phenoxyethanol. The significance of differences between the means was evaluated by Dunnett’s test following the one way analyses of variance (ANOVA); *p < 0.05 versus SAL group; ^#^ *p* < 0.05, ^##^ *p* < 0.01 versus OIL group

Since EE and BE were dissolved and administered in saline solution, whereas PHE in oil, so the results obtained from animals treated with EE and BE were compared to SAL group, while those derived from animals administered PHE to OIL group. All the examined compounds were injected in a dose of 2.5 mmol/kg (s.c.). It was found that 4-week administration of PHE or BE inhibited the total antioxidant activity in both brain regions, e.g. in the frontal cortex and in the hippocampus (Fig. 1a, b). Among tested ethylene glycol ethers, only EE did not change the total antioxidant activity in any of the examined brain regions.

### Lipid Peroxidation

**Fig. 2:**
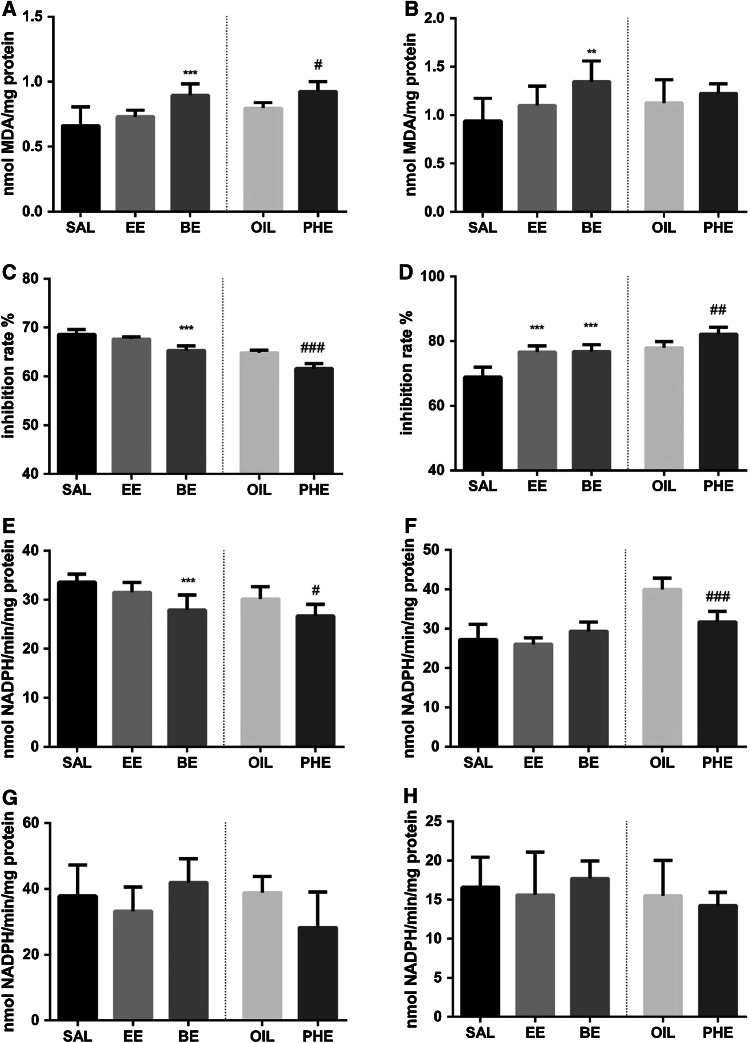
The effect of particular EGEs on the lipid peroxidation in the frontal cortex (**a**) and hippocampus (**b**). On figures **c** and **d** the effect of particular EGEs on the activity of superoxide dismutase is shown in frontal cortex and hippocampus respectively. The effect of particular EGEs on the activity of glutathione peroxidase is shown on figures **e** for frontal cortex and **f** for the hippocampus. The effect of particular EGEs on the activity of glutathione reductase is shown on figures **g** and **h** for frontal cortex and hippocampus respectively. Results are shown as the mean ± SEM from eight tissue samples run in three repetitions. *SAL* saline; *EE* 2-ethoxyethanol; *BE* 2-butoxyethanol; *OIL* sunflower oil; *PHE* 2-phenoxyethanol. The significance of differences between the means was evaluated by Dunnett’s test following the one way analyses of variance (ANOVA); *p < 0.05, **p < 0.01, ***p < 0.001 versus SAL group; ^#^ *p* < 0.05, ^##^ *p* < 0.01, ^###^ *p* < 0.001 versus OIL group

In animals treated with PHE and BE, a significant increase in lipid peroxidation in the frontal cortex was observed (Fig. 2a), whereas in the hippocampus only BE evoked a raise in the MDA concentration (Fig. 2b). There were no significant changes in the lipid peroxidation after exposure animals to EE in both, the hippocampus and frontal cortex.

### Superoxide Dismutase Activity

In animals exposed to BE and PHE, the activity of SOD decreased in the frontal cortex, whereas there was no change in this parameter in rats treated with EE (Fig. 2c). Interestingly, administration of all studied ethylene glycol ethers elevated SOD activity in the hippocampus (Fig. 2d).

### Catalase Activity

It has been found that none of the examined compounds affected in a statistically significant manner catalase activity either in the frontal cortex or in the hippocampus (data not shown).

### Glutathione Peroxidase Activity

In animals treated with PHE and BE, but not with EE, a significant decrease in the activity of glutathione peroxidase in the frontal cortex was observed (Fig. 2e). In the hippocampus only PHE, but not EE and BE, significantly attenuated activity of the investigated enzyme (Fig. 2f).

### Glutathione Reductase Activity

All ethylene glycol ethers under study did no change glutathione reductase activity in the frontal cortex and in the hippocampus (Fig. 2g, h).

## Discussion

The present data indicated that a long term administration of BE and PHE reduced the total antioxidant activity and enhanced lipid peroxidation in the brain. Oxidative stress, resulting from excessive production of free radicals in relation to the capacity of their removal, is considered to be the main cause leading to cell damage. Overproduction of free radicals is particularly dangerous for the central nervous system (CNS) cells, especially for neurons, which as post-mitotic cells are particularly sensitive to the harmful influences. Additionally, in the brain a large amount of free radicals is formed not only as a result of normal aerobic cellular metabolism but also due to metabolism of neurotransmitters, especially excitatory amino acids. Contribution of the oxidative stress to brain damage induced by cerebral ischemia/reperfusion, traumatic brain injury or in pathogenesis of neurodegenerative disorders has often been reported (Sharma et al. 2014; Uttara et al. 2009). There is also a mounting evidence that free radicals play an important role in brain cell damage caused by a number of xenobiotics. Previously, we found that administration of a mixture of EGEs attenuated antioxidant potency in some brain regions, however, because two compounds were administered simultaneously, these data did not provide information, which exactly EGE was responsible for this effect (Pomierny et al. 2013). The present data showed that EGEs with more lipophilic properties, e.g. BE and PHE affected the total antioxidant activity and lipid peroxidation, whereas the most hydrophilic compound—EE showed no such action. The finding that the adverse effects of EGEs on brain cells depends mainly on their lipophilicity is in line with our previous in vitro study, which showed that PHE, BE and IPE much more strongly damaged human neuroblastoma cell line (SH-SY5Y cells) than the hydrophilic EGEs did (Regulska et al. 2010).

To unify dermal passage of active compound through the skin in the present research we administered EGEs by the s.c. injection. The hydrophilic/lipophilic properties of EE, BE, PHE are versatile, thus dermal administration could have distracted absorption rate of particular EGEs. Effectiveness of s.c. route of administration was confirmed in the research of similar group of EGEs (Starek et al. 2008a) and stress induced by this method of administration was negligible.

The aim of the present study was also to find out whether changes in the total antioxidant activity and lipid peroxidation in animals receiving PHE and BE were associated with differences in the activity of antioxidant enzymes present in these brain structures. Enzymatic and non-enzymatic antioxidants are responsible for scavenging free radicals, their precursors or inhibiting ROS production mainly by binding metal ions involved in their synthesis. Among enzymatic antioxidants, catalase, SOD and GPX play an essential role in the initial stage of ROS removal. In the frontal cortex, the decrease in the total antioxidant activity, was accompanied by the reduction in SOD and GPX activity. SOD catalyzes dismutation of superoxide radical to hydrogen peroxide, which is next detoxified by GPX and catalase. Thus, the reduction of SOD activity induced by PHE and BE may be responsible for toxic effect of superoxide radicals, while the decrease in GPX can potentiate hydrogen peroxide-induced damage. We did not observe any changes in catalase and glutathione reductase activities but due to a relatively low level of enzymatic antioxidants in the brain and high susceptibility of this tissue to damage, it seems that the decrease only in SOD and GPX may be sufficient to evoke adverse effects in the frontal cortex. Accordingly, enhancement of lipid peroxidation, one of the first signs of free radical action on polyunsaturated fatty acids in biological membranes, was observed in the frontal cortex in animals administered BE and PHE. The obtained results clearly show that in the frontal cortex PHE and BE, but not EE, reduce ability of enzymatic antioxidants to scavenge free radicals. In contrast to the frontal cortex, in the hippocampus the reduced total antioxidant activity in animals administered PHE and BE was not connected with the reduction of the investigated antioxidant enzyme activities. All examined EGEs increased SOD activity and had no effect on catalase and GR. Only PHE significantly attenuated GPX activity but this effect did not intensify lipid peroxidation. On the other hand, BE which both reduced the total antioxidant activity and increased lipid peroxidation, did not reduce the activity of any of the investigated enzymes and even enhanced SOD activity. Thus, oxidative damage of lipids induced by this compound probably resulted from its action on other enzymes involved in free radical removal (e.g. glutathione S-transferase) or on non-enzymatic antioxidants (e.g. ascorbic acid, *α*-tocopherol, carotenoids). Most likely is the impact of BE on vitamin D since it has been shown that this compound strongly reduces vitamin E level in the peripheral tissue (Park et al. 2002), however, such effect in the brain has not been studied, yet. It is also possible that SOD activation by BE and PHE may accelerate superoxide radical conversion to hydrogen peroxide, which under constant catalase activity may not be sufficiently decomposed to H_2_O_2_ that altogether leads to an increase in the hydroxyl radicals formation.

Like in the present study, also attenuation of antioxidant defense system and increase in lipid peroxidation appears to be the mechanism of damaging EGE action on testicular function (Adedara and Farombi 2010). Similarly, it has been shown that BE administration induced oxidative stress in the mouse liver and probably by this mechanism BE increased liver tumor (Park et al. 2002). In that case, BE increased oxidative stress in an indirect way, namely it induced hemolysis which led to the accumulation of hemosiderin and free hemoglobin in the liver, next iron in Fenton reaction exacerbated the formation of hydroxyl radicals and induced liver neoplasia. However, the mechanism of EGE action in the brain has not been studied and it cannot be concluded whether the exacerbation of lipid peroxidation, observed in the present study, resulted from the direct action of EGEs on antioxidant enzymes or it was an indirect action, connected for instance with increase in the ROS production, blocking metabolic cooperation between brain cells or decrease in intracellular pH (Loch-Caruso et al. 1984; Louisse et al. 2010). It has not been also established whether neurotoxic EGE action is exerted by the parent compound or as in the peripheral tissues by their metabolites, mainly alkoxyacetic acids. Currently, we believe that the neurotoxic effects of these compounds depend mainly on their lipophilicity which determines their penetration to the brain. However, this assumption is not supported by determination of the level of these compounds and their metabolites in CNS.

In conclusion, the present study indicated that the most lipophilic EGEs induced oxidative stress and enhanced lipid peroxidation in the rat frontal cortex and hippocampus. In the frontal cortex, but not in the hippocampus, the adverse effects of EGEs were likely to be connected with inhibition of SOD and GPX activity. Thus, excessive exposure to EGEs can induce or exacerbate brain cell damage and consequently these compounds may play a role in pathogenesis of neurodegenerative disorders.
